# Supplementary material for: Modeling glioblastoma heterogeneity as a dynamic network of cell states
Source: Mol Syst Biol. 2021 Sep 16;17(9):e10105. doi: 10.15252/msb.202010105 (PMC8444284; doi:10.15252/msb.202010105)
Supplement: Supplementary file 5 — Source Data for Figure 3 [file MSB-17-e10105-s001.zip › Figure3A_sourcedata/GSEA_3065/nosigdb_state2.GseaPreranked.1622718419215/NB_KI_CLUSTER18.html]

Details for gene set NB\_KI\_CLUSTER18[GSEA]

|  || Dataset | state2 |
| Phenotype | NoPhenotypeAvailable |
| Upregulated in class | na\_neg |
| GeneSet | NB\_KI\_CLUSTER18 |
| Enrichment Score (ES) | -0.7896003 |
| Normalized Enrichment Score (NES) | -2.8938115 |
| Nominal p-value | 0.0 |
| FDR q-value | 0.0 |
| FWER p-Value | 0.0 |
Table: GSEA Results Summary

  

Fig 1: Enrichment plot: NB\_KI\_CLUSTER18      
 Profile of the Running ES Score & Positions of GeneSet Members on the Rank Ordered List

  

| PROBE | GENE SYMBOL | GENE\_TITLE | RANK IN GENE LIST | RANK METRIC SCORE | RUNNING ES | CORE ENRICHMENT || 1 | PDLIM1 |  |  | 452 | 0.190 | -0.0309 | No |
| 2 | MT2A |  |  | 731 | 0.146 | -0.0472 | No |
| 3 | TPM4 |  |  | 848 | 0.133 | -0.0475 | No |
| 4 | YBX3 |  |  | 1677 | 0.078 | -0.1287 | No |
| 5 | ZFP36L1 |  |  | 2320 | 0.053 | -0.1924 | No |
| 6 | LY6E |  |  | 2588 | 0.045 | -0.2168 | No |
| 7 | LIMA1 |  |  | 3279 | 0.028 | -0.2878 | No |
| 8 | JUNB |  |  | 3923 | 0.017 | -0.3549 | No |
| 9 | UACA |  |  | 3925 | 0.017 | -0.3535 | No |
| 10 | SERTAD1 |  |  | 4915 | 0.001 | -0.4590 | No |
| 11 | CEBPB |  |  | 5541 | -0.009 | -0.5248 | No |
| 12 | SOCS3 |  |  | 6224 | -0.020 | -0.5958 | No |
| 13 | CYTOR |  |  | 6306 | -0.022 | -0.6024 | No |
| 14 | LHFPL6 |  |  | 6336 | -0.022 | -0.6035 | No |
| 15 | SH3BGRL |  |  | 6546 | -0.026 | -0.6234 | No |
| 16 | VAMP5 |  |  | 6835 | -0.032 | -0.6512 | No |
| 17 | MEF2C |  |  | 6861 | -0.033 | -0.6508 | No |
| 18 | PHLDA1 |  |  | 6867 | -0.033 | -0.6483 | No |
| 19 | NOTCH3 |  |  | 7196 | -0.042 | -0.6795 | No |
| 20 | FOS |  |  | 7222 | -0.043 | -0.6783 | No |
| 21 | TIMP3 |  |  | 7309 | -0.045 | -0.6834 | No |
| 22 | RGS16 |  |  | 7474 | -0.050 | -0.6963 | No |
| 23 | STOM |  |  | 7570 | -0.052 | -0.7017 | No |
| 24 | A2M |  |  | 7912 | -0.063 | -0.7323 | No |
| 25 | TPM2 |  |  | 8019 | -0.068 | -0.7374 | No |
| 26 | THY1 |  |  | 8139 | -0.074 | -0.7434 | No |
| 27 | MFGE8 |  |  | 8573 | -0.101 | -0.7803 | Yes |
| 28 | FILIP1L |  |  | 8586 | -0.102 | -0.7723 | Yes |
| 29 | LMNA |  |  | 8636 | -0.106 | -0.7679 | Yes |
| 30 | C1R |  |  | 8644 | -0.107 | -0.7589 | Yes |
| 31 | TAGLN |  |  | 8662 | -0.108 | -0.7509 | Yes |
| 32 | LGALS1 |  |  | 8680 | -0.110 | -0.7427 | Yes |
| 33 | VIM |  |  | 8691 | -0.110 | -0.7337 | Yes |
| 34 | FCGRT |  |  | 8702 | -0.111 | -0.7246 | Yes |
| 35 | CST3 |  |  | 8712 | -0.112 | -0.7153 | Yes |
| 36 | IFITM3 |  |  | 8720 | -0.113 | -0.7058 | Yes |
| 37 | TGFBI |  |  | 8761 | -0.117 | -0.6993 | Yes |
| 38 | MYH9 |  |  | 8778 | -0.119 | -0.6902 | Yes |
| 39 | SELENOM |  |  | 8855 | -0.129 | -0.6865 | Yes |
| 40 | MYLK |  |  | 8862 | -0.131 | -0.6751 | Yes |
| 41 | CYR61 |  |  | 8901 | -0.136 | -0.6668 | Yes |
| 42 | COL18A1 |  |  | 8909 | -0.137 | -0.6551 | Yes |
| 43 | TNFRSF1A |  |  | 8962 | -0.143 | -0.6476 | Yes |
| 44 | PRSS23 |  |  | 8975 | -0.145 | -0.6356 | Yes |
| 45 | AXL |  |  | 9023 | -0.152 | -0.6267 | Yes |
| 46 | SERPINH1 |  |  | 9054 | -0.159 | -0.6154 | Yes |
| 47 | TFPI |  |  | 9060 | -0.159 | -0.6014 | Yes |
| 48 | COL4A2 |  |  | 9069 | -0.161 | -0.5875 | Yes |
| 49 | CAVIN1 |  |  | 9071 | -0.161 | -0.5730 | Yes |
| 50 | COL6A2 |  |  | 9116 | -0.170 | -0.5621 | Yes |
| 51 | CAV1 |  |  | 9130 | -0.172 | -0.5478 | Yes |
| 52 | CD99 |  |  | 9224 | -0.203 | -0.5392 | Yes |
| 53 | CEBPD |  |  | 9235 | -0.205 | -0.5216 | Yes |
| 54 | GSN |  |  | 9238 | -0.206 | -0.5030 | Yes |
| 55 | ID3 |  |  | 9252 | -0.214 | -0.4849 | Yes |
| 56 | COL4A1 |  |  | 9284 | -0.226 | -0.4676 | Yes |
| 57 | RRBP1 |  |  | 9287 | -0.227 | -0.4470 | Yes |
| 58 | IGFBP7 |  |  | 9295 | -0.229 | -0.4269 | Yes |
| 59 | CDKN1A |  |  | 9301 | -0.230 | -0.4064 | Yes |
| 60 | LGALS3 |  |  | 9308 | -0.236 | -0.3855 | Yes |
| 61 | SPARCL1 |  |  | 9360 | -0.288 | -0.3647 | Yes |
| 62 | FN1 |  |  | 9364 | -0.294 | -0.3381 | Yes |
| 63 | SPARC |  |  | 9384 | -0.332 | -0.3099 | Yes |
| 64 | TIMP1 |  |  | 9387 | -0.334 | -0.2796 | Yes |
| 65 | ITGB1 |  |  | 9388 | -0.339 | -0.2486 | Yes |
| 66 | CALD1 |  |  | 9391 | -0.348 | -0.2170 | Yes |
| 67 | CD63 |  |  | 9394 | -0.353 | -0.1850 | Yes |
| 68 | COL6A1 |  |  | 9410 | -0.410 | -0.1492 | Yes |
| 69 | IGFBP5 |  |  | 9438 | -0.797 | -0.0794 | Yes |
| 70 | NEAT1 |  |  | 9439 | -0.871 | 0.0001 | Yes |
Table: GSEA details [plain text format]

  

Fig 2: NB\_KI\_CLUSTER18: Random ES distribution      
 Gene set null distribution of ES for **NB\_KI\_CLUSTER18**
